# Decreased *AGO2* and *DCR1* in PBMCs from War Veterans with PTSD leads to diminished miRNA resulting in elevated inflammation

**DOI:** 10.1038/tp.2017.185

**Published:** 2017-08-29

**Authors:** M Bam, X Yang, E E Zumbrun, J P Ginsberg, Q Leyden, J Zhang, P S Nagarkatti, M Nagarkatti

**Affiliations:** 1 Department of Pathology, Microbiology and Immunology, University of South Carolina School of Medicine, Columbia, SC, USA; 2 William Jennings Bryan Dorn VA Medical Center, Columbia, SC, USA; 3 Division of Virology, USAMRIID, Fort Detrick, MD, USA; 4 Department of Epidemiology and Biostatistics, Arnold School of Public Health, University of South Carolina, Columbia, SC, USA

## Abstract

Chronic inflammation is a characteristic of post-traumatic stress disorder (PTSD). The initiation of inflammation and molecules involved are not yet clearly understood. Here, we provide compelling evidence that the inflammation seen in PTSD may result from the dysregulated miRNA processing pathway. Using microarray analysis with a discovery group of peripheral blood mononuclear cell (PBMC) samples from War Veterans with PTSD, we found 183 significantly downregulated miRNAs, several of which target numerous genes categorized to be pro-inflammatory in nature. This observation was further confirmed in a replicate group by including more samples. Furthermore, employing RNA-sequencing, quantitative real time PCR (qRT-PCR) and *in vitro* experiments, we found that Argonaute 2 (*AGO2*) and Dicer1 (*DCR1*) were downregulated in PTSD and provided convincing evidence that their downregulation affects mature miRNA generation. In addition, we noted that *STAT3* transcript was reduced in PTSD and this was possibly responsible for reduced *AGO2* and *DCR1*, which in turn affected miRNA synthesis. Furthermore, we observed that activation of CD4+ T cells or monocytes led to reduced mature miRNA availability. Finally, the inflammation seen in PTSD was associated with downregulated miRNA profile. Altogether, the current study demonstrates that the chronic inflammation seen in PTSD may be a result of dysregulated miRNA biogenesis pathway due to diminished expression of the key molecules like *AGO2*, *DCR1* and *STAT3*.

## Introduction

Exposure to a traumatic event such as military combat, violence and natural disasters, can lead to the development of post-traumatic stress disorder (PTSD).^1^ The prevalence of PTSD is estimated to be ~3.5%^2^ in the U.S. Following combat, the rate increases significantly to ~20% in U.S. military service personnel,^3^ leading to an estimated annual healthcare cost of around 180 million USD.^4^ The quality of life of the patient is significantly affected as the symptom of PTSD may be prolonged. As per DSMV-IV classification, symptoms of PTSD include hyperarousal, intrusive thoughts, flashbacks, nightmares, numbing of feelings, insomnia, fear, avoidance of reminders, irritability, hypervigilance, heightened startle response and distress when exposed to reminders.^5^ It is now clear that PTSD is a serious psychiatric disorder associated with a dysregulated immune system. Differential expression of several pro-inflammatory genes and dysregulated networks has already been reported in PTSD in the recent past.^6, 7, 8, 9, 10^ Moreover, in a recent review, after analyzing published literature on immune status, the authors concluded that PTSD patients exhibit an excessive inflammatory state.^11^ Above all, PTSD has also been associated with other clinical disorders such as cardiovascular disease, diabetes, gastrointestinal disease, fibromyalgia, chronic fatigue syndrome, musculoskeletal disorders and autoimmune diseases,^12, 13^ all of which have an inflammatory component.

We have reported elevated expression of pro-inflammatory cytokines in PTSD and provided evidence linking this with dysregulated expression of miRNAs and other epigenetic marks. Zhou *et al.*^7^ reported that pro-inflammatory cytokines such as interferon gamma (IFNG) and interleukin (IL) 17 were significantly elevated in PTSD and their expression was inversely related to miR-125a. In another study from our lab, Bam *et al.*^8^ reported that the level of IL12 was elevated in PTSD due to decreased expression of miR-193a-5p. These reports suggested that in PTSD, there was dysregulation in expression of miRNAs that control the expression of pro-inflammatory cytokines. When considering dysregulation of miRNAs, involvement of a faulty miRNA biogenesis mechanism must be considered. In this context, deficiency in DCR1, the enzyme responsible for the generation of mature miRNA by cleaving pre-miRNA to ~22 nucleotide mature miRNAs, is known to result in deficient miRNA biogenesis and can lead to lowered abundance of mature miRNAs.^14, 15, 16^ Remarkably, a recent report showed that the expression of Dicer1 was decreased in PTSD, and this was linked to the lowered expression of miR-3130-5p.^17^ Interestingly, the authors reported only miR-3130-5p as significantly downregulated but did not show any link between the downregulated miRNA and any physiological changes in PTSD. Recently, the AGO2-dependent pathway, which is independent of the DCR1-dependent pathway, for miRNA biosynthesis has been documented in the mammalian system.^18, 19, 20, 21^ As an example, miR-451, which controls erythropoiesis, was shown to be exclusively processed through the AGO2-dependent miRNA biogenesis pathway.^20^

Gene expression can be regulated at various stages including transcription, post transcription, translation and post-translation.^22, 23, 24^ In response to signal from cytokines and growth factors, specific signal transducer and activator of transcription (STAT) protein members are phosphorylated and translocated to the nucleus where they activate target gene transcription.^25, 26, 27^ As analyzed by Genomatix Pathway System (GePS, a tool for predicting transcription factors (TFs) available at www.Illumina.com), STAT3 was predicted to influence the transcription of *AGO2* and *DCR1*, apart from several other TFs. However, it is not yet known whether STAT3 interacts directly with the promoter of *AGO2* and *DCR1* or, the influence on transcription is mediated indirectly.

In the current study, we investigated miRNA biogenesis and found that miRNA biogenesis key molecules, *AGO2* and *DCR1*, were downregulated along with the expression of a large number of miRNAs in the peripheral blood mononuclear cells (PBMCs) of War Veterans suffering from PTSD. On the basis of our data, we suggest that the inflammation seen during PTSD is probably initiated due to lowered expression of the molecules that regulate mature miRNA generation.

## Materials and methods

### Patients

Informed and written consent was obtained from all participants included in the study. PTSD patients were Veterans of either the 1991 Persian Gulf war, or of the recent Iraq or Afghanistan wars, recruited from William Jennings Bryan Dorn Veterans Medical Center, as described earlier.^6^ All of the donors were first clinically assessed by professionals for PTSD. Participants were evaluated by the psychometric properties of the PTSD Checklist (PCL)^28^ and the PTSD diagnosis was validated by the Clinician Administered PTSD Scale^29^ and the Diagnostic and Statistical Manual of Mental Disorder (DSM-V).^4^ PTSD patients with current alcohol and other substance abuse, undergoing immunosuppressive drug treatment or having immunosuppressive disease, were excluded. For normal controls, age-matched healthy volunteers were included. Individuals who did had symptoms of active infection or history of immune compromise such as HIV, cancer, pregnancy or were on chronic steroid therapy, were not included for the study.

We included (a) 4 controls vs 8 PTSD patients in the discovery group and 7 controls vs 3 PTSD in the replication group for the miRNA array analysis; (b) 5 controls and 5 PTSD patients for the RNA-sequencing (RNA-seq) analysis and (c) 22 controls and 18 PTSD patients for all the quantitative real time PCR (qRT-PCR) validation studies. The demographic of the samples included for microarray and RNA-Seq technologies are provided in Bam *et al.*^10^

### Sample collection and RNA isolation

Peripheral blood samples (10–20 ml) were collected in EDTA coated collection tubes (BD Biosciences, San Jose, CA, USA) and PBMCs were isolated using Ficoll-Paque (GE Healthcare, Uppsala, Sweden) within 1 h of sample collection. PBMC viability was determined by trypan-blue exclusion. Using a universal kit (AllPrep DNA/RNA/miRNA Universal Kit, Qiagen, Valencia, CA, USA) recommended for simultaneous isolation of high quality DNA and total RNA including miRNAs, all of the entities were isolated from the same ~10 million PBMCs and immediately frozen at −80 °C until use. All our PBMC samples were de-identified/blinded to us.

### Micro-RNA microarray

Microarray for the miRNAs was performed at Johns Hopkins Memorial Institute (Deep Sequencing and Microarray Core Facility, Baltimore, MD, USA). Total RNA, including mRNA, miRNA and other small RNA molecules, were isolated from PBMC samples as described above. Next, total RNA samples were used in the analysis of miRNA expression level by miRNA array hybridization assay using the Affymetrix miRNA-v1 gene chip (Affymetrix, Sunnyvale, CA, USA). Linear fold-change in miRNA was calculated to compare the differences of all the miRNAs expressed between PTSD patients and controls. A linear fold-change of at least ±1.5 was used as a cutoff value for the inclusion of a miRNA. Moreover, only the miRNAs which were significant on the basis of *P-*value (<0.05) calculated using student’s *t*-test, were included for the analysis. For analyzing the miRNAs in the replication group, we combined all the data from both discovery and replication group. The miRNA array data are available in ArrayExpress (EMBL-EBI, Cambridgeshire, UK, Accession# E-MTAB-4880).

### RNA-seq

RNA-Seq libraries were constructed using Illumina TruSeq RNA Sample Preparation kit (Illumina, San Diego, CA, USA). Briefly, total RNA was purified from PBMCs using the Qiagen RNA easy kit. The oligo-dT beads were added to 1 μg of total RNA to isolate mRNA. The purified mRNA was fragmented to 200–400 bases. The RNA fragments were then reverse transcribed into double stranded cDNA fragments. The DNA fragments were repaired to generate blunt ends using T4 DNA polymerase, Klenow polymerase and T4 polynucleotide kinase. After DNA fragments were purified using Qiagen PCR purification kit (Qiagen #28004), an ‘A’ base was added to the 3′ end of the blunt DNA fragment by Klenow fragment. Sequencing adapters were ligated to the ends of DNA fragments using DNA ligase. The libraries were then amplified by limited PCR (15 cycles) using primers provided by the kit. The PCR products were then separated by 2% agarose gel electrophoresis and fragments with sizes ranging from 250 to 400 bp were excised and purified using the QIAquick Gel Extraction Kit (Qiagen #28704). The concentration and distribution of the library were determined by a NanoDrop spectrophotometer (Thermo Scientific, Wilmington, DE, USA). The library was sequenced by Illumina HiSeq 2000 at Tufts University Genomic core facility. Raw sequencing reads (50 bp single-end) were mapped to human genome build hg19 using Tophat 2.^30^ The accepted hits were used for assembling transcripts and estimating their abundance using Cufflinks. The differentially expressed gene, promoter usage and splicing form were determined by Cuffdiff and Cuffcompare.^31^ The heat maps and links were generated using Circos software.^32^ Our data are available in NCBI’s GEO database (Accession #GSE83601).

### Levels of precursor miRNAs

The expression level of miRNA precursors can be detected by performing comparison of microarray data, next generation sequencing (NGS) data or by employing qRT-PCR analysis for individual precursors.^33^ We compared the expression of precursor (pri- and pre-miRNAs) miRNAs between controls and PTSD patients by looking into the NGS data. Because precursors are longer in length, these molecules are detected by RNASequencing. We compared the precursor levels of only the miRNAs listed in Figure 2e. The difference in the expression of the precursors was expressed as fold-change. The fold-change and the statistical significance values were obtained after analyzing the RNA-Seq data in Cufflink and Cuffdiff.

### qRT-PCR analysis

All qRT-PCR reactions were performed in an Applied Biosystem ViiA 7 Real-Time PCR system (Life Technologies, ThermoFisher Scientific, Waltham, MA, USA). From all the samples, cDNA was synthesized after taking 1 μg each of total RNA in a 20 μl system using miScript II RT kit from Qiagen (Qiagen) following manufacturer instructions. For all the samples, the quantity and purity of total RNA was analyzed by Nanodrop (Thermo Scientific) equipment. For the detection of gene transcripts, 5–10 ng of cDNA per well in a 10 μl system of a 384 well were used and amplified with iQ universal SYBR Green supermix (Bio-Rad, Hercules, CA, USA). As an internal control, 18S rRNA and GAPDH message was quantified along with the genes. The expression level of the genes was expressed as the relative abundance (RE) value to the internal control. For the detection of miRNAs, cDNA was synthesized as mentioned above and 4ng was used in each well of a 384 well plate in a 10 μl per well system. We used miScript SYBR Green PCR Kit (Qiagen) and miScript Primer Assay (Qiagen) for the qRT-PCR detection of the respective miRNAs. Furthermore, we selected eight genes (JAK2, STAT1, IL23A, TGFB1, TGFB2, TGFB3, T-BET and CXCL3) predicted or known to be targeted by miRNAs found downregulated in our data set. The primer sequences are provided in Table 1.

### siRNA-based knockdown of AGO2 and STAT3

siRNA for respective targets were designed using the BLOCK-iT software (ThermoFisher Scientific) and the oligos were procured from Integrated DNA Technologies (Coralville, IA, USA). Sequences of the siRNAs are provided in Table 1. To knock down the target genes, THP-1 cells were seeded at 2 × 10^5^ cells in complete RPMI 1640 (with β-mercaptoethanol) 24 h prior to transfection in 24 well plates. For the transfection, a final amount of 15 pmol of siRNA per well was used and transfection performed using Lipofectamine RNAi MAX (Life Technologies, ThermoFisher Scientific) following the instructions from the manufacturer. In the cultures with a time period exceeding 48 h, 350 μl medium was replaced at 48 h. All the cell types were cultured with 5% CO_2_ at 37 °C. After the indicated culture time, cells were harvested and total RNA isolated using the AllPrep DNA/RNA/miRNA Universal Kit (Qiagen). RNA samples were stored at −80 °C for future use.

### Isolation and activation of CD4+ T cells

CD4+ T cells were isolated from PBMCs collected fresh from healthy individuals using EasySep kit (StemCell Technologies, Cambridge, MA, USA) and PE-conjugated mAb (Cat #555347, BD Biosciences) against CD4+ cells following the manufacturer’s instructions. The purity of the isolated CD4+ T cells was confirmed by running an aliquot in a Flow Cytometer (FC500, Beckman Coulter, Indianapolis, IN, USA). For the activation of the CD4+ T cells, 1x10^6^ cells were taken in 1000 μl of complete RPMI medium in a 24 well plate and 25 μl Dynabeads Human T-Activator CD3/CD28 (1:1 bead-to-cell ratio) (Life Technologies, ThermoFisher Scientific, Cat. #111.61D), 30 U human rIL-2 (Biolegend, San Diego, CA, USA) and cultured the cells in 5% CO_2_ at 37 °C for 48 h. After the culture periods, cells were collected and total RNA was isolated.

### Stimulation of THP-1 cells

To stimulate THP-1 cells, purified lipopolysaccharide, from *E. coli* at 1 μg ml^−1^ and recombinant human IFNG @ 100ng ml^−1^ was used with 1x10^6^ THP-1 cells in complete RPMI medium and cultured for 24 and 48 h in a 5% CO_2_ at 37 °C. After the indicated culture periods, cells were collected and total RNA isolated as described earlier. Our cell line was obtained from ATCC and was authenticated recently at University of Arizona Genetics Core.

### miRNA-target gene prediction

Ingenuity Pathway Analysis (IPA, Qiagen, Redwood City, CA, USA) has the ability to provide information on targets of miRNAs for a data set. We used this tool to predict the targets of the differentially expressed miRNAs in PTSD. Toward this end, we used the 190 differentially expressed miRNAs and included some pro-inflammatory genes, as shown in the interactive network, and connected them by the IPA algorithm. IPA will connect the miRNA and the target gene only if they are experimentally shown and reported or predicted on the basis of computational analysis.

### Prediction of STAT3-targeted genes

We identified the probable genes that could be a binding site for STAT3 by using the Genomatix Pathway System tool available with Illumina. This tool identifies genes with promoter having a binding sequence of a transcription factor, which is based on the available evidence from published literatures.

### Statistics

For obtaining the *P-*values, we performed the Student’s two tailed *t-*tests and Wilcoxon and Mann–Whitney tests. All the *in vitro* experimental results are from multiple (three or more) replicates.

According to the previous study, we assumed the group standard deviation as 0.4. Then, with a sample size of 4 controls and 8 patients for the microarray, it is adequate to obtain at least 80% power to detect around 100 genes with a true difference in expression of at least 1.5 with a false discovery rate (FDR) of 0.05 using a two-sided two-sample *t*-test. Thus, the power was adequate when it was combined with the replication data.

## Results

### miRNA and long non-coding RNA expression profiles in PBMCs of PTSD patients

**Figure 1:**
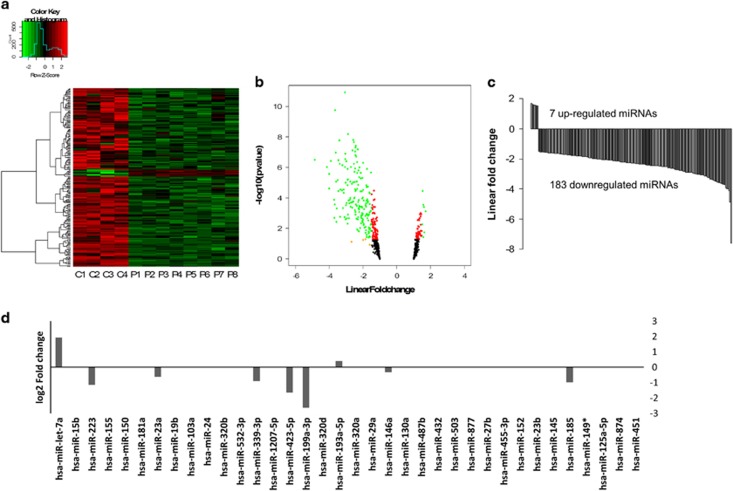
miRNA expression profile in PBMCs from post-traumatic stress disorder (PTSD) patients. (**a**) Microarray analysis of miRNAs in PBMCs from normal human controls and PTSD patients. The heat map shows the expression intensities of all the miRNAs in each of the control and PTSD samples after microarray. (**b**) The volcano plot shows all the miRNAs with at least ±1.5 linear fold-difference. The green dots represents miRNAs significantly dysregulated in PTSD patients. (On *y* axis, 1.301 units is ~0.05). A total of 190 miRNAs were found to be significantly (*P-*value<0.05) up- (7 miRNAs) or downregulated (183 miRNAs) by a linear fold-change value of ±1.5 or more (**c**). (**d**) The expression levels of the precursors of miRNAs as obtained from RNA-Seq analysis and presented here in the figure as fold-change values. The names represent the corresponding precursors of the miRNAs. These 35 miRNAs were further analyzed in the *in vitro* studies. PBMC, peripheral blood mononuclear cell.

Employing microarray, we analyzed miRNA expression in the PBMCs obtained from 8 PTSD patients and compared it with 4 controls that did not experience trauma. Only miRNAs with a linear fold-change value of at least ±1.5 and *P-*value <0.05 were considered for further analysis. Using these criteria, a total of 190 mature miRNAs were obtained, of which 183 were downregulated and only 7 were upregulated (Figure 1a–c). These data were interesting in suggesting that most of the miRNAs are downregulated in the PBMCs of PTSD patients.

Because we were focusing on mature miRNA biogenesis, we compared the levels of precursors. The names of the nucleotide sequences are provided as NCBI Accession numbers in the RNA-Seq result after analysis in Cufflink and Cuffdiff. Thus, individual accession numbers were matched with the miRNA precursor by confirming the names in HUGO Gene Nomenclature Committee website. We observed that there was no significant difference in the levels of the precursors after RNA-Seq analysis (Figure 1d). The RNA-Seq data showed that 9 of the 35 precursors had some difference in the degree of their expression in PTSD. However, none of the differences were statistically significant.

Realizing the interesting potential role played by long non-coding RNAs (lncRNAs), we studied the expression pattern of lncRNAs in our RNA-Seq data. We found 39 lncRNAs that were significantly altered in their expression levels in PTSD compared with the controls. Of the 39 significant lncRNAs, 25 (64%) were downregulated and rest upregulated in PTSD patients. The most highly up- and downregulated ones were NR_001434/HLA-H lncRNA (3.5 log_2_ fold-change) and NR_037194/ LINC00664 (−3.7 log_2_ fold-change), respectively. Table 2 provides the list of significantly dysregulated lncRNAs and their differential expression levels as fold-change values.

### Expression of AGO2 and DCR1 transcripts in the PBMCs of PTSD patients is lowered

**Figure 2:**
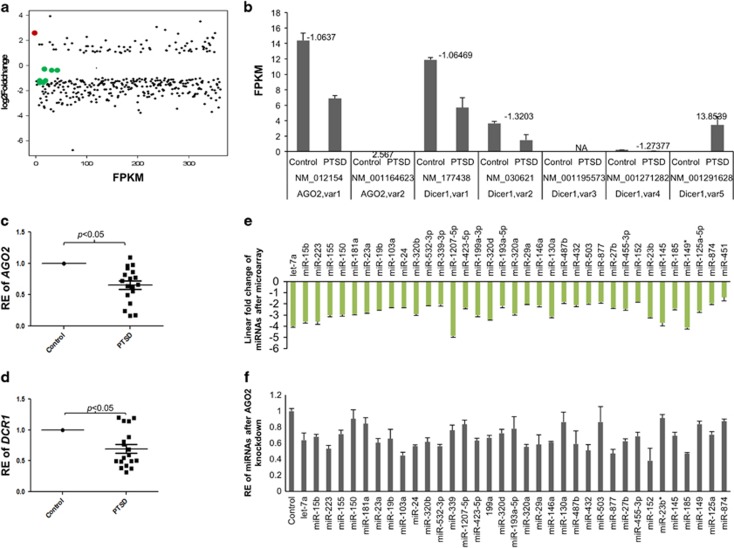
Argonaute 2 (*AGO2*) and Dicer1 (*DCR1*) transcript is lower in PBMCs of post-traumatic stress disorder (PTSD) patients and the abundance of mature miRNAs is reduced upon decreased expression of AGO2 and DCR1. (**a**) RNA-Seq analysis expression values of only the genes significantly dysregulated with log_2_ fold-change of at least 1 or more (readers are requested to refer Bam *et al.*^10^ for the complete list of the genes), and different variants of *AGO2*, *DCR1* and *STAT3*. The green dots indicate the position (expression values) of the different variants of *AGO2*, *DCR1* and *STAT3*. The red dot is the position of *DCR1* variant 5. (**b**) The FPKM values of the variants of *AGO2* and *DCR1* in control and PTSD samples after RNA-sequencing (RNA-Seq) analysis. The values above the bars indicate log_2_ fold-change when compared between PTSD and controls. (FPKM: Fragments per kilobase of transcript per million mapped reads). (**c**, **d**) Quantitative real time PCR validation result of *AGO2* and *DCR1* transcripts in 22 controls and 18 PTSD PBMC RNA samples. The difference in expression level is provided as relative expression (RE) value by taking the controls as 1. In this assay, 18S rRNA was used as an internal control. (**e**) Linear fold-change values of 35 miRNAs, after microarray analysis, which were selected for further analysis by *in vitro* experiments. miRNA-451 was also included in the figure as a positive control because it was reported previously to be processed specifically through the AGO2-dependent pathway. (**f**) Relative expression levels of 34 miRNAs, listed in Figure 3a, after knockdown of AGO2 for 72 h by employing siRNA in THP-1 cells. The expression level is expressed relative to control which was taken as 1. PBMC, peripheral blood monnuclear cell.

Mature miRNA generation requires cleavage by DCR1 of the pre-miRNA. Moreover, it has been reported recently that AGO2-dependent miRNA biogenesis is another mechanism to generate mature miRNAs independent of DCR1. Thus, to understand why most mature miRNAs were downregulated in PTSD, we first asked whether AGO2 and/or DCR1 are altered in the PBMCs of PTSD patients. We performed RNA-seq analysis with total RNA from PBMCs of PTSD and control samples and detected that both *AGO2* and *DCR1* were downregulated in the PBMCs of PTSD patients when compared with controls (Figures 2a and b). To corroborate the RNA-seq results, we analyzed the transcript levels of both *AGO2* and *DCR1* employing qRT-PCR by including 22 control and 18 PTSD samples. As seen in Figures 2c and d, both the genes were significantly downregulated in PTSD. These data indicated that there is alteration in the miRNA biogenesis pathway involving AGO2 and DCR1 in the PBMCs of PTSD patients.

### Reduced AGO2 results in decreased miRNA abundance

In as much as *AGO2* and *DCR1* were also downregulated parallel to downregulated miRNAs (Figure 2e), we next tested whether downregulation of AGO2 can lead to downregulated expression of miRNAs. It is known that lowered expression of DCR1 can lead to lowered abundance of mature miRNAs. However, the same is not fully understood for AGO2. Thus, to answer the above question, we knocked down AGO2 using siRNAs and measured the abundance of miRNAs. As shown in Figure 2f, after 72 h knockdown of AGO2, several miRNAs were downregulated in THP-1 cells. It should be noted here that not all miRNAs analyzed were found to be downregulated after knockdown of AGO2. It is possible that the miRNAs that were not downregulated are independent of AGO2 processing pathway.

### AGO2 and DCR1 expression is positively affected by STAT3

**Figure 3:**
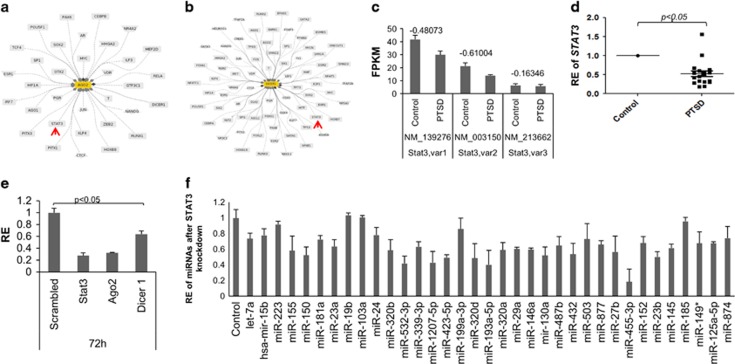
*STAT3* expression is lowered in the PBMCs of post-traumatic stress disorder (PTSD) patients. (**a**, **b**) Network showing predicted transcription factors for AGO2 and DCR1. (**c**) Transcript level of the three reported *STAT3* variants in the PBMCs of PTSD patients as analyzed by RNA-seq (the values above the bars indicate log_2_ fold-change). (**d**) Relative abundance of *STAT3* transcripts in the PBMCs of PTSD patients after analysis by qRT-PCR with 22 control and 18 PTSD samples. Here, 18S rRNA was used as an internal control. (**e**) Relative abundance of *AGO2, DCR1* and *STAT3* transcripts 72 h post-knockdown of STAT3. (**f**) STAT3 was knocked down using siRNA in THP-1 cells and miRNAs were quantified after 72 h. The figure shows RE of miRNAs after knockdown of STAT3. (RE: relative enrichment). PBMC, peripheral blood mononuclear cell.

Next, we investigated whether there is a common factor that could influence expression of both *AGO2* and *DCR1* together. We searched for transcription factors that could interact with both *AGO2* and *DCR1* promoters, using the Genomatix Pathway System tool available with Illumina. As shown in Figures 3a and b, respectively, STAT3 was predicted to interact with both *AGO2* and *DCR1* promoters as a transcription factor. It should be noted that there are other transcription factors predicted to interact with both *AGO2* and *DCR1* promoters. However, none of them, by RNA-Seq analysis, had all of the transcript variants dysregulated like *STAT3*. To that end, we checked the level of expression of *STAT3* transcripts in our RNA-Seq data and observed that all the three variants of *STAT3* were downregulated in the PBMCs of PTSD patients compared with healthy controls (Figure 3c). This observation was further confirmed in a larger sample size by analysis of 22 controls and 18 PTSD samples by qRT-PCR (Figure 3d).

Next, we investigated whether lowered expression of STAT3 would affect the transcription of *AGO2* and *DCR1*. For this, we knocked down STAT3 using siRNA in THP-1 cells and measured *AGO2* and *DCR1* by qRT-PCR analysis. We observed that after 72 h of knocking down STAT3, the transcript level of both *AGO2* and *DCR1* were significantly reduced (Figure 3e). These data indicated that STAT3 regulates the expression of both *AGO2* and *DCR1* possibly as a transcription factor or an important subunit of a complex that regulates the transcription of both the genes.

### Mature miRNA abundance is lowered with reduced STAT3

Next, we investigated whether lowering STAT3 affects the abundance of mature miRNAs. To that end, we analyzed the abundance of 35 miRNAs in the STAT3 knockdown samples. We observed that majority of the miRNAs were less abundant after 72 h of knocking down of STAT3 (Figure 3f). This observation further implied that STAT3 may regulate the expression of both *AGO2* and *DCR1*, because of which there is diminished expression of the miRNAs with lowered STAT3 expression.

### Activation of CD4+ T or THP-1 cells show evidence of lowering the abundance of miRNAs

**Figure 4:**
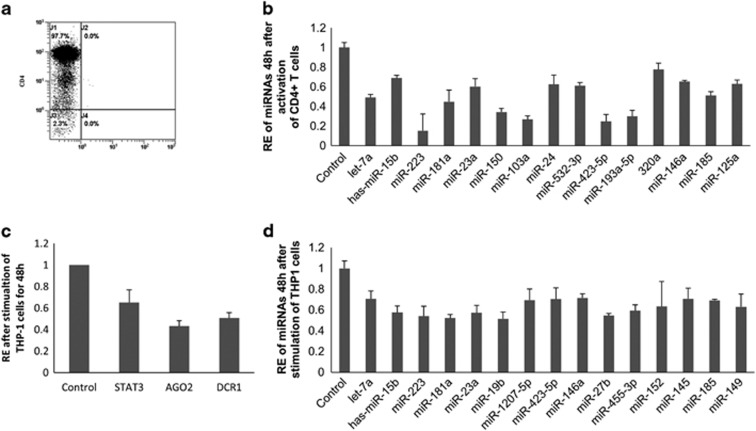
Cell activation leads to changes in the abundance of mature miRNAs. (**a**) Purity of CD4+ T cells after isolation from PBMCs of a healthy control. CD4+ T or THP-1 cells were activated/stimulated and miRNA levels were quantified after 48 h. (**b**) Relative expression levels of the listed miRNAs in CD4+ T cells after activation for 48 h. (**c**) Levels of *STAT3*, *AGO2* and *DCR1* transcripts after stimulation of THP-1 cells for 48 h. Transcript levels of all the three genes were significantly lesser compared to control. (**d**) miRNA relative expression levels are indicated after stimulation of THP-1 cells for 48 h.

We were interested in seeing the effect of the activated state of CD4+ T cells in the generation of miRNAs because we have reported earlier that PTSD patients have more activated CD4+ T cells.^7^ To that end, we isolated CD4+ T cells from PBMCs of a healthy donor, with >97% purity, as analyzed by flow cytometry (Figure 4a). Post-activation of the CD4+ T cells, miRNAs were measured after 48 h (Figure 4b) and we observed that the majority of the miRNAs from the total of 35 analyzed were lowered in their abundance as compared to control, upon activation of CD4+ T cells.

Because PBMCs include monocytes, we were interested in studying the effect of stimulation/activation of these cells on the expression of miRNAs. To that end, we used THP-1 cells, a cell line established from human monocytes and found, post-stimulation with lipopolysaccharide and IFNG, decreased levels of *STAT3*, *AGO2* and *DCR1* (Figure 4c). Also, we observed that majority of the miRNAs from our list of selected miRNAs were lowered in their abundance after 48 h, as compared to that of control (Figure 4d).

### Genes targeted by downregulated miRNAs are involved in inflammation

**Figure 5:**
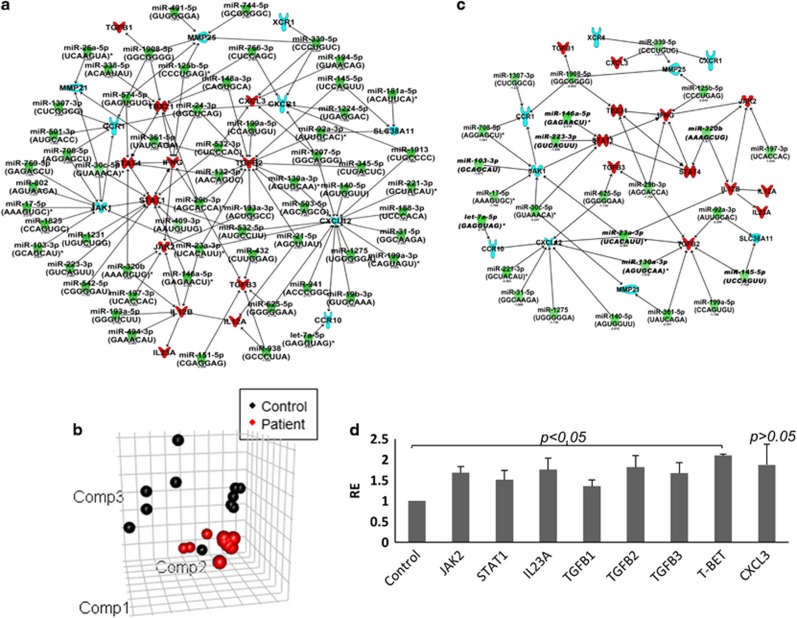
miRNAs downregulated in post-traumatic stress disorder (PTSD) target several pro-inflammatory genes. (**a**) Ingenuity Pathway Analysis (IPA) was performed with the dysregulated miRNAs in PTSD. The genes were included in the network and the miRNAs were connected by the IPA algorithm. The network shows direct interaction (predicted and proven) between miRNAs and target genes as indicated by solid arrows. (**b**) Principal component analysis (PCA) plot showing the relatedness of the samples. (**c**) IPA-generated network showing the interaction between miRNAs and targets after analyzing with the miRNAs obtained in the replicate samples. The red and blue colored target genes in the network correspond to up- and downregulated, respectively, validated for its expression level and reported either in the current manuscript or in our previous reports with studies on the same samples. For the miRNAs, the red color indicates upregulated and green indicates downregulated as per microarray analysis. (**d**) To validate the transcript levels of miRNA-targeted genes, qRT-PCR was performed to detect transcript of eight genes as shown in the graph (genes were selected based on the miRNA–gene interaction networks). The *P-*values of the genes under the curly brackets were <0.05.

To gain further insight into the underlying mechanism leading to heightened inflammation during PTSD, we analyzed the dysregulated miRNAs (190) observed in PTSD samples and found their predicted or known target genes. We used the Qiagen miRNA-target interactive network designer tool within IPA to obtain the predicted or known targets of the dysregulated miRNAs in our data set. The claim made on the basis of this analysis is strongly supported by our previous reports on elevated pro-inflammatory cytokines in PTSD as a result of lowered miRNA expression targeting specific genes recognized as pro-inflammatory in nature. Figure 5a demonstrated that several pro-inflammatory genes are either known or predicted to be targets of numerous miRNAs that are downregulated in PTSD. For example, IFNG and IL12 are pro-inflammatory cytokines produced by various cells upon activation (chiefly by CD4+ T cells and monocytes/macrophages, respectively). As another example, T-BET (TBX21) is the major transcription factor that controls expression of IFNG and was also a target of several downregulated miRNAs. As mentioned previously, we have already reported the elevated expression of the genes mentioned above. To enhance the reliability of our observations, in the current manuscript, we further confirmed the dysregulated miRNA expression in a replication group where we included more samples for the microarray analysis. As seen in Figure 5b, the PTSD samples in the replication group distinctly separated from the controls in a principal component analysis plot analysis. In the replication group, we obtained 7 up- and 77 downregulated miRNAs, which had similar expression profiles as seen in the discovery group. Upon miRNA–gene interaction analysis with the dysregulated miRNAs obtained from the replication group, we observed that several of the miRNAs that targeted the same pro-inflammatory genes overlap between the two groups (Figure 5c). To further corroborate our observations, we performed qRT-PCR validation of 8 genes (JAK2, STAT1, IL23A, TGFB1, TGFB2, TGFB3, T-BET and CXCL3) predicted to be the targets of the downregulated miRNAs (Figure 5d). The analysis showed that the transcript levels of all the genes listed above were found to be significantly (except CXCL3) higher in PTSD patients compared with controls.

## Discussion

PTSD is a psychiatric disorder associated with a chronic inflammatory condition. The precise mechanisms that trigger inflammation during PTSD remain unclear. In this study, we provide new and interesting data which demonstrate a global defect consisting of decreased expression of *AGO2* and *DCR1*, which in turn downregulates the expression of a large number of miRNAs in PTSD. This observation provides a clear indication of a possible deficiency in the mature miRNA biogenesis pathway. DCR1 is the main catalytic enzyme that cleaves the pre-miRNA to generate the mature miRNA.^34, 35^ Thus, it is expected that decreased expression of *DCR1* would lead to decreased generation of mature miRNAs and thereby cause lowered abundance of miRNAs as seen in this study with PTSD.

On the basis of the previous reports on AGO2-dependent, DCR1-independent, mature miRNA generation,^18, 19, 20, 21^ it can be suggested that lowered *AGO2* expression can also result in decreased abundance of mature miRNAs. However, there is no distinction of miRNAs based on whether they are generated through DCR1 or AGO2-dependent pathways, except for a few such as miR-451.^20^ Our observation is consistent with this study demonstrating that miR-451 was significantly downregulated in the PBMCs of PTSD patients. However, besides miR-451, we do not know which of the differentially expressed miRNAs from our data set are selectively processed through the AGO2-dependent pathway. To prove that lowered *AGO2* affects miRNA biogenesis, we knocked down *AGO2* and observed a decrease in expression of some of the miRNAs we selected to study. We believe these miRNAs could be processed by the AGO2-dependent pathway, further implying that lowered *AGO2* can affect the biosynthesis of miRNAs. We also observed that the abundance of some of the miRNAs were similar to that of control indicating that AGO2 was not the only miRNA biogenesis pathway involved.

To rule out that the observed difference in mature miRNA level in PTSD was not because of lowered transcription levels of the miRNA genes, we compared the expression levels of the pri- and pre-miRNAs. The pri- and pre-miRNAs are the two preceding stages of a mature miRNA in the miRNA biogenesis pathway. Considering that the transcription of the miRNA genes are equal in controls and PTSD, a bias in the processing of the precursors can lead to differential levels of the mature miRNAs. Our NGS data indicated that the level of precursors is more or less equal in controls and PTSD patients. This is indicative of an equal degree of transcription of miRNA genes in both controls and PTSD. However, as we have mentioned earlier, the altered levels of the corresponding mature miRNA in PTSD would imply that there is indeed a bias in the processing of the miRNA precursors after its gene is transcribed at an equal degree. Thus, this observation could be further linked to a biased pri-/pre-miRNA processing machinery in PTSD leading to an overall decreased level of mature miRNAs. Our knockdown experiments also indicated that lowering of either AGO2 or DCR1 can lead to a lowered level of the mature miRNA.

The next question we addressed was whether there was a common factor that regulates the expression of *AGO2* and *DCR1*. To that end, we used the Genomatix Pathway System tool and searched for transcription factors that could interact with both *AGO2* and *DCR1* promoters, and identified STAT3 to potentially interact with the promoters of both *AGO2* and *DCR1*. When the level of STAT3 was knocked down, both the expression of *AGO2* and *DCR1* were also decreased thereby implying that their expression was regulated probably at the transcription level, by STAT3. Corroborating this observation, *STAT3* was significantly downregulated in the PBMCs of PTSD patients clearly indicating that the downregulation of *AGO2* and *DCR1* could be the result of reduced expression of *STAT3*. We do not know why *STAT3* is downregulated in the PTSD PBMCs, but we hypothesize that this in turn causes decreased transcription of both *AGO2* and *DCR1*, which directly diminish the generation of mature miRNAs and the resultant effect is observed as a global downregulation of miRNAs in PTSD PBMCs. In support of this, many of the miRNAs selected for analysis were also reduced in their abundance after *in vitro* knockdown of *STAT3*. Thus, in the light of the recent report by Wingo *et al.*,^36^ we believe that indeed DCR1 and AGO2 are downregulated in the PBMCs of PTSD patients, which possibly results from decreased STAT3 expression, and eventually culminate into a lowered miRNA expression. Thus, although our findings related to STAT3 expression indicated that it is having a pro-inflammatory role in PTSD, it should be noted that STAT3 can have an anti-inflammatory role as well depending on the signaling molecule. It is known that in the presence of signaling from IL10/IL10R, the downstream signaling via STAT3 results in an anti-inflammatory state.^37^ Because in our present study, we do not know the status of IL10, it is not possible to make a concluding remark. Moreover, the very few available reports on IL10 level in PTSD is not convincing.^38, 39, 40, 41, 42, 43^ Nevertheless, it appears that IL10 expression level is more in individuals who are resilient to PTSD development.^44^ Therefore, it further supports our hypothesis that STAT3 is probably having a pro-inflammatory role in PTSD.

As our previously published reports suggested activated state of T cells and monocytes/macrophages in PTSD, we wanted to see whether there is any link between the activated state of T cells and monocytes and diminished miRNA expression. We found that the abundance of some of the miRNAs is lowered in activated CD4+ T and THP-1 cells. This observation indicates that activated state of CD4+ T cells or monocytes might be possibly linked to lowered miRNA expression in the PBMCs of PTSD patients. Moreover, we found that *STAT3*, *AGO2* and *DCR1* were also lowered upon stimulation of THP-1 cells. This observation implies that mature miRNA abundance could also be possibly lowered since the key molecules involved in their synthesis are lowered. Furthermore, it also implies that, at least in THP-1 cells (may be applicable to monocytes as well), stimulation can lead to lowering of miRNA biosynthesis. However, as only some of the miRNAs were downregulated upon activation/stimulation, it will be interesting to test whether the reduction of miRNAs is the cause for the activation of the cells leading to a pro-inflammatory state or vice versa in PTSD patients.

We have previously reported the link between lowered miRNA level and elevated pro-inflammatory cytokines in PTSD. The bioinformatics tool-based analysis clearly indicated that numerous significantly downregulated miRNAs in PTSD target classical pro-inflammatory cytokines and transcription factors. These observations imply that regulation of the target genes at the post-transcriptional stage could be inefficient. In such a state, there will be more target genes (mRNA) available for translation resulting in elevated level of the proteins. Furthermore, in the present study, we found that many of the pro-inflammatory genes like JAK2, STAT1, CXCL3, IL23A and so on, were found to be significantly upregulated in PTSD patients. This observation further hints that indeed there is a deficiency in the regulation of the expression of genes in PTSD. In our study, it appears that the downregulated miRNA expression leads to increased level of target gene transcripts. Thus, it is possible that the inflammation seen during PTSD is a result of differentially regulated pro-inflammatory genes due to a failure in the gene expression regulation involving miRNAs, apart from other regulatory mechanisms like DNA methylation and histone modifications.^6, 8, 10, 45, 46^ It is important to note that the interaction between miRNA and mRNA leads to destabilization/degradation of the mRNA in 66 to >90% of the instances.^47^ Therefore, we hypothesize that as a result of lowered abundance of miRNAs, the regulation of target genes at the post-transcriptional level by miRNAs is inefficient, which leads to an elevated expression of pro-inflammatory genes resulting in the observed inflammation in PTSD patients.

lncRNAs are gaining significant attention recently due to their not yet clearly understood ways of functioning in the control of gene expression. The lncRNAs are considered a new class of gene regulators with various unknown mechanisms of action. These are a class of non-protein coding RNA molecules found to be expressed in different cell types.^48, 49, 50, 51^ In the recent past, it has been demonstrated that lncRNAs are critical regulators of gene expression, including immune system-related genes.^52, 53, 54, 55, 56^ However, because there is no significant literature available yet to support the role of these lncRNAs in our data set, we have not discussed all of these. Nevertheless, a few from our list of dysregulated lncRNAs are reported in literature. For example, LUCAT1, LINC01002 and MIR3945HG (all were downregulated in our data set) were shown to be dysregulated and believed to have a role in lung cancer.^57, 58, 59, 60, 61^ As another example, TPT1-AS1 (upregulated in our data set) was reported to be associated with the prognosis of anaplastic gliomas.^62^ On a different note, interestingly, we observed that the expression pattern of the significantly dysregulated lncRNAs also had a similar pattern as the miRNA expression in PTSD. We saw that there was more downregulated lncRNAs in PTSD compared with controls. We do not know whether there is any significance attached to this observation. However, we believe that this aspect of the observation is definitely a promising area to look into in the future.

In summary, our present data demonstrate that the inflammatory status seen in PTSD is due at least in part to the lowered abundance of miRNAs, which may result from reduced levels of *AGO2* and *DCR1* resulting in inefficient biosynthesis of mature miRNAs that target pro-inflammatory genes. Furthermore, the lowered expression of *AGO2* and *DCR1* is probably due to reduced expression of *STAT3* in PBMCs of PTSD patients. However, it is not clear from our studies whether miRNA downregulation is the result of activated state of the cells or downregulation of miRNAs leads to activated state of the cells of the immune system. A deeper study in the miRNA biosynthesis pathway in a similar setting as ours can definitely lead to a better understanding of the precise role of the key molecules that can be used in the development of effective therapeutic and/or management strategies for PTSD in War Veterans and the general population.

## Figures and Tables

**Table 1:** Sequence details of the primers for the qRT-PCR, and the siRNAs used for the knockdown experiments

*Primers*	*Sequence*
* JAK2 f*	tgggcagaattagcaaacct
* JAK2 r*	tgtgtaggatcccggtcttc
* STAT1 f*	tgaagacaggggtccagttc
* STAT1 r*	agactgccattggtggactc
* IL23A f*	agcttcatgcctccctactg
* IL23A r*	ttagggactcagggttgctg
* TGFB1 f*	cacgtggagctgtaccagaa
* TGFB1 r*	tgcagtgtgttatccctgct
* TGFB2 f*	ttgacgtctcagcaatggag
* TGFB2 r*	ttcgccttctgctcttgttt
* TGFB3 f*	aactggctgtctgccctaaa
* TGFB3 r*	tatagcgctgtttggcaatg
* T-BET f*	cccaccatgtcctactaccg
* T-BET r*	gcaatctcagtccacaccaa
* CXCL3 f*	gcagggaattcacctcaaga
* CXCL3 r*	ggtgctccccttgttcagta
* STAT3 f*	cagggtgtcagatcacatgg
* STAT3 r*	aaggtgcctggaggcttagt
* DCR1 f*	aggatgaggaggaggagagc
* DCR1 r*	tttgggcattttccattcat
* AGO2 f*	tcgcactatcacgtcctctg
* AGO2 r*	atggcttccttcagcactgt
	
*siRNA*	
* AGO2* sense	GCAGAAACACACCUACCUU
* AGO2* antisense	AAGGUAGGUGUGUUUCUGC
* STAT3* sense	GCGUCCAGUUCACUACUAA
* STAT3* antisense	UUAGUAGUGAACUGGACGC

Abbreviations: f, forward; r, reverse.

**Table 2:** Differentially expressed lncRNAs in PTSD detected after RNA-Seq analysis

*Gene id*	*log_2_ (fold-change)*	*Gene id*	*log_2_ (fold-change)*	*Gene id*	*log_2_ (fold-change)*
NR_037194	−3.69867	NR_002189	−1.8776	NR_027353	1.12582
NR_110907	−3.33775	NR_109769	−1.77106	NR_033841	1.17126
NR_026817	−3.0604	NR_036534	−1.70274	NR_024433	1.29705
NR_103549	−2.76247	NR_103791	−1.54662	NR_027293	1.35455
NR_037142	−2.59941	NR_103718	−1.45414	NR_024458	1.44115
NR_126161	−2.55986	NR_003187	−1.37966	NR_036466	1.6418
NR_028324	−2.38937	NR_028502	−1.32709	NR_030732	1.77503
NR_027072	−2.25576	NR_120420	−1.31823	NR_026958	1.89413
NR_103548	−2.17617	NR_003186	−1.31434	NR_028062	2.18775
NR_027256	−2.11981	NR_003505	−1.2379	NR_038929	2.18956
NR_047572	−1.97086	NR_039983	−1.16287	NR_038402	2.24131
NR_037867	−1.93293	NR_104116	−1.06893	NR_047651	2.57877
NR_126168	−1.90112	NR_040085	1.06824	NR_001434	3.52181

Abbreviation: PTSD, post-traumatic stress disorder. The names/ids are the NCBI accession numbers and the values indicate the expression difference in fold-change.
